# Kawasaki Disease: A Clinician's Update

**DOI:** 10.1155/2013/645391

**Published:** 2013-10-27

**Authors:** Nathan Jamieson, Davinder Singh-Grewal

**Affiliations:** ^1^School of Women's and Children's Health, University of New South Wales, Sydney, NSW 2052, Australia; ^2^The Sydney Children's Hospitals Network, Randwick and Westmead Campuses, Sydney, NSW, Australia

## Abstract

*Aims*. Kawasaki disease is an acute systemic vasculitis and is the most common cause of acquired heart disease in children in the developed world. This review aims to synthesise recent insights into the disease and provide an update for clinicians on diagnostic and treatment practices. *Methods*. We conducted a review of the literature exploring epidemiology, aetiology, diagnosis, and management of Kawasaki disease. We searched MEDLINE, Medline In-Process, Embase, Google Scholar, and reference lists of relevant articles. *Conclusions*. Kawasaki disease is a febrile vasculitis which progresses to coronary artery abnormalities in 25% of untreated patients. The disease is believed to result from a genetically susceptible individual's exposure to an environmental trigger. Incidence is rising worldwide, and varies widely across countries and within different ethnic groups. Diagnosis is based on the presence of fever in addition to four out of five other clinical criteria, but it is complicated by the quarter of the Kawasaki disease patients with “incomplete” presentation. Treatment with intravenous immunoglobulin within ten days of fever onset improves clinical outcomes and reduces the incidence of coronary artery dilation to less than 5%. Given its severe morbidity and potential mortality, Kawasaki disease should be considered as a potential diagnosis in cases of prolonged paediatric fever.

## 1. Introduction

Kawasaki disease (KD) is an acute systemic vasculitis which progresses to cause coronary artery abnormalities in 25% of untreated patients [1]. It has surpassed rheumatic heart disease as the leading cause of acquired cardiovascular disease in children in the developed world [2]. Though over 40 years have passed since its initial identification in patients by Kawasaki [3], its aetiology remains largely unknown and a specific diagnostic test eludes researchers and clinicians. Diagnosis is based primarily on clinical criteria; however, the presentation is incomplete in approximately 25% of patients, and this patient subset appears to be at a comparatively higher risk of serious complications [4]. Coronary artery dilation and aneurysms can be prevented by timely administration of intravenous immunoglobulin, a limited and costly resource, placing an onus on practitioners to diagnose the disease promptly and accurately.

## 2. Epidemiology

Kawasaki disease was shown to account for 23% of all paediatric vasculitides in a United States rheumatology clinic population study and is the second most common multisystem vasculitis of infancy and childhood behind Henoch-Schönlein purpura [5–7]. Though significant differences in epidemiological distribution have been observed worldwide [8], a number of factors appear relatively constant. These include a male predominance, with a male-to-female ratio of between 1.5 : 1 and 2 : 1 [9–11]; marked seasonality, with heightened incidence in winter and early spring in temperate climates [12] and summer peaks in some Asian countries [13] approximately 75% of cases occurring in children under 5 years of age [10, 14]; and a heightened incidence in people of Asian descent, both inside and outside Asia [15–17]. A rising incidence has been observed worldwide over time, perhaps due to heightened awareness and recognition of the disease [8, 10, 14, 18].

## 3. Aetiology

Determining the exact nature of the causative mechanism behind KD remains an area of controversy, unresolved since Kawasaki first described the condition in 1967 [3]. It is hypothesised that KD results from the exposure of a genetically predisposed individual to an as-yet unidentified, possibly infectious environmental trigger [6]. As such, the discussion of potential genetic and environmental aetiologies should be viewed as complementary rather than conflicting theories.

### 3.1. Genetic Aspect of Aetiology

The epidemiology of the condition suggests that Mendelian inheritance is not at play in determining KD susceptibility. Epidemiological data is strongly suggestive of a genetic component to aetiology, with studies worldwide reporting focal heightened incidence in children of East Asian origin. While the highest incidence of KD is observed in Japan, this incidence is matched in North America by Japanese-American children, with an incidence of 210.5/100,000. This trend is also present in children of Pacific Islander, native Hawaiian, and Korean descent [15, 19, 20]. A genetic predisposition is also supported by the higher relative risk of KD within families, whereby siblings of a KD patient are at a 10-fold higher risk of KD compared to the general population [16, 21].

Linkage analysis and genome-wide association studies have identified several single nucleotide polymorphisms that show an association with genetic susceptibility to KD. Two prominent discoveries include functional polymorphisms of the FC gamma RIIa locus and the ITPKC gene, which have been shown to predispose individuals in Japan and North America to KD and the subsequent development of coronary artery aneurysms [16, 22, 23]. The ITPKC gene is a T-cell activity modulator, and its identification as a susceptibility gene suggests that T-cell activity regulation may be an underlying mechanism in determining predisposition to and severity of the disease course [24]. 

### 3.2. Environmental Aspect of Aetiology

It is widely postulated that, following exposure to one or multiple environmental triggers in childhood, the immune response in a small, genetically susceptible patient subset manifests as systemic vasculitis [18]. This hypothesis is supported by the abrupt symptomatic onset of KD, the seasonal and temporal clustering of cases, the spontaneous resolution of the disease even without treatment in the majority of cases, and the predomination of IgA plasma cells at mucosal surfaces in the immune response, common characteristics of the infectious diseases of childhood [13, 22, 25, 26]. Additionally, KD occurs principally between the ages of three months and five years, when susceptibility to ubiquitous infectious agents is at its highest. This epidemiological clustering suggests that most adults have immunity to the causative agent following exposure, and transplacental antibodies protect newborn infants [17, 18, 27, 28]. 

It is possible that bacterial or viral infections, superantigens, humoral factors, or a combined superantigen-conventional peptide antigen response may underlie the onset of the disease [29, 30], though to date, no aetiological agents have been confirmed in studies [6]. Up to 33% of KD patients have at least one concurrent infection at the time of diagnosis, but no correlation between a specific agent and the severity of the disease course has been identified [31]. Though the clinical and biochemical similarities seen in KD and staphylococcal and streptococcal toxin-mediated illnesses are suggestive of the involvement of a bacterial toxin in the disease aetiology, Rowley et al. [32] have isolated intracytoplasmic inclusion bodies in the ciliated bronchial epithelium of acute-stage KD sufferers, suggesting instead that an intracellular viral pathogen is likely to be at work. An intrinsic autoimmune cause seems unlikely based on current evidence, given patients' lack of autoantibodies and the spontaneous resolution and absence of recurrence seen in the condition [24].

## 4. Diagnosis 

### 4.1. Clinical Course

The clinical course of KD consists of four phases: (1) acute, the period lasting 1-2 weeks if untreated, when the child has a spiking, often remittent 40° Celsius fever and principal symptomatic features and may present with cardiac manifestations including valvitis, pericarditis, and myocarditis; (2) subacute, the approximately 2 week period following the abatement of fever when the child is at the greatest risk of sudden death due to myocardial infarction; (3) convalescent, the clinically invisible period following the cessation of symptoms and continuing until acute-phase reactants return to normal serum levels; and (4) chronic, which describes patients who require follow-up management due to coronary artery involvement [11, 18, 33–35]. Diagnosis should occur in the acute stage so that prompt treatment can be administered to abate inflammation and reduce the risk of coronary artery involvement in the later disease phases [2]. 

### 4.2. Diagnosis of Acute KD

There is no specific diagnostic test available for KD to date [36]. Diagnosis is currently based on clinical criteria, as established by the Japanese Ministry of Health and adopted by the American Heart Association [2] and is presented in Table 1 alongside the nonspecific laboratory tests used to support the diagnosis. Neither the clinical criteria nor laboratory features have been systematically validated as diagnostic tools [37], and the clinical criteria rely on the recognition of nonspecific symptoms that may not be present in KD but can be present in a number of other vasculitides, toxin-mediated illnesses, viral exanthema, and inflammatory conditions [29]. Differential diagnoses for KD include Epstein-Barr virus, adenovirus, echovirus, measles, toxic shock syndrome, scarlet fever, idiopathic juvenile arthritis, polyarteritis nodosa, Rocky Mountain spotted fever, leptospirosis, juvenile mercury poisoning, and adverse drug reactions, including Stevens-Johnson syndrome [29, 38, 39].

This dependence on clinical criteria is problematic, given that the features of KD may manifest sequentially rather than simultaneously [40]. The principal clinical findings can be accompanied by the multitude of symptoms that accompany febrile vasculitides, including arthritis, gastrointestinal upset, rhinorrhoea, weakness, extreme irritability, hydrops of the gallbladder, and mild anterior uveitis, all of which may contribute to misdiagnosis and treatment delay [2, 41]. Additional occasionally seen features such as pyuria and pleocytosis of the cerebrospinal fluid can suggest that other infectious processes underlie the presenting complaint and delay diagnosis [42]. This variability in patient presentation places pressure on clinicians to consider KD in any case of prolonged and otherwise unexplained fever.

Clinical diagnosis is hindered further by the significant subset of patients who present with incomplete KD, characterised as KD wherein less than four of the principal features are present but laboratory results or echocardiography suggests the diagnosis of KD. Patients less than 12 months old and children older than five are more likely to present with incomplete KD [37, 46]. These patients, who make up approximately 25% of the KD cohort [4], are at a heightened risk of experiencing coronary artery complications because the associated higher rates of misdiagnosis may delay appropriate treatment, an issue compounded by their placement outside the typical KD age cohort [40, 47, 48].

### 4.3. Diagnostic Procedure for Cardiovascular Sequelae

Two-dimensional echocardiography is fundamental in the assessment of the coronary arteries following the diagnosis of KD based on the presence of the principal clinical features, or if KD is suspected in a patient that otherwise does not fulfill the diagnostic criteria. Echocardiography is a sensitive and specific means of detecting dilation, ectasia, or aneurysms in children [47], though the use of mild sedative agents may be required in younger children to ensure that the quality of the echocardiographic imaging is not compromised by excessive movement. Abnormal echocardiographs may show ectasia, defined by a body-adjusted *Z* score of the dilation of the left anterior descending or right coronary artery of 2.5 or greater, or by the presence of a segmental aneurysm [2].

Aneurysms in children are classified by the American Heart Association as small (5 mm internal diameter), medium (5–8 mm internal diameter), or giant (8 mm internal diameter). The Japanese Ministry of Health criteria for assessing the coronary arteries are age dependent and state that coronary arteries should be considered abnormal if the internal lumen diameter measures greater than 3 mm in children under 5 years or greater than 4 mm in children of 5 years, if the internal diameter of a segment is 1.5 times the diameter of an adjacent segment, or if obvious lumen irregularities are present [2]. Giant coronary aneurysms are the least likely to regress and have the highest association with progression to stenosis and myocardial infarction [49]. Regression is facilitated by smaller aneurysm size, fusiform morphology, and location in a distal coronary segment [11].

## 5. Acute Phase Management

The aim of acute phase management in KD is to reduce inflammation, particularly coronary arteritis and myocarditis [50]. Though the mechanisms underlying KD are not completely understood and the efficacy of intravenous immunoglobulin (IVIG) as a first-line treatment in acute-phase KD has been validated in a number of prospective multicentre treatment trials [51, 52]. The American Heart Association recommends that patients be treated with a single infusion of IVIG over twelve hours at a dosage of 2 g/kg within ten days of fever onset, along with an anti-inflammatory 100 mg/kg/day dose of aspirin (acetylsalicylic acid) spread out over 4 doses until the child is afebrile [2]. A recent Japanese randomised control trial found that the addition of 2 mg/kg/day prednisolone to the standard IVIG regimen significantly reduced adverse coronary outcomes, though these results have yet to be replicated in non-Japanese populations [53].

While aspirin is a standard component of the treatment regime worldwide, evidence as to its benefit is sparing. To date, no sufficiently-powered randomised control trials have proven the benefit of aspirin versus placebo, though it is believed to be of benefit given its antiplatelet and anti-inflammatory effects at high doses [8, 54]. However, concerns about the risks of high-dose aspirin administration, including aspirin toxicity, Reye's syndrome, and sensorineural hearing loss, have led to adjustments to dosing practices in some countries including Japan, where the recommended acute-phase dose has been lowered to 30–50 mg/kg/day [55].

Timely administration of treatment is critical in preventing adverse coronary artery outcomes. IVIG administered ≥ten days after fever onset facilitates a reduction in inflammation but is ineffective in preventing coronary artery lesions [56]. Studies have also reported a higher rate of IVIG resistance if patients are treated before the fifth day of fever, though it is unclear whether early treatment leads to poorer outcomes or if patients that present with KD before the fifth day have a more severe form of the disease [36].

### 5.1. IVIG Resistance

Between 11% and 23% of patients may present with IVIG resistance, diagnosed if a patient exhibits persistent or recurrent fever at least 36 hours after the first IVIG dose has been infused [57, 58]. IVIG resistance is problematic because recalcitrant fever is indicative of ongoing arteritis, which places patients at a higher risk of developing coronary artery aneurysms [39]. It is recommended that refractory disease is first treated with a second dose of IVIG 2 g/kg, though the efficacy of a number of other therapeutic options, including intravenous corticosteroid pulse therapy, anti-TNF-alpha antibodies, and cytotoxic agents, is an ongoing area of research [8, 11, 59].

### 5.2. Aneurysm Management

Aneurysm management in the acute phase of KD is an area of uncertainty, and it is recommended that a paediatric cardiologist be involved in patient care and the development of an individualised action plan if echocardiography shows dilation or aneurysm of the coronary arteries after diagnosis. The presence of coronary artery dilation necessitates the early involvement of a paediatric cardiologist, frequent echocardiography to monitor the coronary arteries, and long-term regular stress and perfusion tests of the heart [27, 60]. 

Percutaneous coronary intervention procedures are indicated in patients at high risk of ischaemia. These procedures include intracoronary thrombolysis, balloon angioplasty, stent implantation, and rotational ablation and should be performed on patients presenting with either symptomatic ischaemia, laboratory findings that suggest ischaemia, or severely stenotic lesions that appear likely to progress to coronary artery ischaemia [43, 60]. Coronary artery bypass surgery is indicated if angiography detects severely occlusive lesions or jeopardised collateral blood supply [60, 61].

## 6. Chronic Phase Management

The aim of chronic phase management is to prevent coronary artery occlusion and myocardial infarction through reducing platelet aggregation and inhibiting thrombogenesis [50, 60]. Long-term treatment consists of antiplatelet aspirin doses of 3–5 mg/kg/day until a normal echocardiograph is seen at 6–8 weeks [18]. If coronary artery abnormalities fail to regress by this time, long-term pharmacological therapy and diagnostic followup are implicated (Table 2).

Patients with coronary artery involvement require long-term antiplatelet doses of aspirin. In addition to this, systemic anticoagulation therapy with warfarin or low molecular weight heparin (LMWH) is indicated in patients with giant or multiple large aneurysms. LMHW is more likely to contribute to a statistically signification reduction in the coronary artery *Z* score and is less likely to result in severe bleeding, making it a viable option for young patients with severe coronary artery involvement [62]. Children who have experienced Kawasaki disease and acute phase coronary artery involvement should reduce exposure to atherosclerosis risk factors, including obesity, hyperlipidaemia, and smoking [60].

It is important that immunisations be delayed in children who have received treatment with IVIG, as this treatment blocks the acquisition of active immunity by preventing the replication of live viral vaccines. Official recommendations state that immunisations should be delayed by 11 months [2]. Inactive immunisations are unaffected by serum IVIG [63, 64]. 

## 7. Clinical Outcomes

Possible outcomes for KD include (1) resolution without cardiac sequelae; (2) the development of coronary artery abnormalities, of which approximately 60% regress in a year; (3) cardiac involvement, including myocarditis, aneurysm thrombosis, cardiac dysrhythmia, or myocardial infarction; and (4) KD recurrence in 3% of patients [1, 65, 66].

Prior to the discovery of IVIG as a safe and efficacious treatment, 20–30% of cases progressed to coronary artery dilation, with a 2% mortality rate [67, 68]. If treated with IVIG within ten days of fever onset, transient coronary artery dilation only develops in 3–5% of children and giant aneurysms develop in 1% [68]. Factors that predispose KD patients to cardiovascular sequelae include a longer duration of fever prior to treatment, low serum albumin upon hospital admission (<3 g/L) [9, 69], age under 1 year or over 5 years [29, 70, 71], and presentation with IVIG resistance [71] or incomplete KD [72].

A conceivable long-term implication for otherwise healthy KD survivors is the potential for accelerated atherosclerosis development. Only a small number of postmortem cadavers have been available for study, though it appears that endothelial dysfunction and coronary artery scarring are present in patients with coronary artery involvement. Despite the presence of histopathological abnormalities upon biopsy of the myocardium, long-term contractility appears normal in patients affected by transient myocarditis during KD [2]. However, increased arterial stiffness secondary to fibrotic changes within the arterial wall is indicative of an increased risk of developing atherosclerosis, which will likely become apparent as the first cohort of identified KD patients approaches middle age [52, 73]. 

## 8. Conclusion 

KD is a disease associated with significant morbidity and potential mortality, yet no specific diagnostic test is available. Increased physician awareness of the principal features of KD and appropriate use of echocardiography have together improved patient outcomes through facilitating timely treatment, but incomplete presentations complicate diagnosis and are associated with significantly worse coronary outcomes. Given the severe consequences of late diagnosis, the immediate benefits offered by available therapies including IVIG, and the rising incidence of KD worldwide, it is important that neonatal and paediatric clinicians consider KD as a diagnosis in cases of prolonged paediatric fever.

## Figures and Tables

**Table 1:** Clinical diagnosis criteria as determined by the American Heart Association and laboratory criteria that may be used to help establish the diagnosis.

American Heart Association diagnostic clinical criteria [2]	Supplementary laboratory criteria [2, 34, 36, 41, 43]
Fever persisting >5 days, plus at least four out of five of the following principal features* (i) Changes in extremities, including indurative angioedema and desquamation (ii) Polymorphous exanthema (iii) Bilateral bulbar conjunctival injection without exudate (iv) Changes to the lips and oral cavity, including pharyngeal injection, dry fissured lips, and/or strawberry tongue (v) Acute nonpurulent cervical lymphadenopathy (>1.5 cm diameter)	(i) Albumin <3 g/dL (ii) C-reactive protein >3 mg (iii) Erythrocyte sedimentation rate >40 mm/h (iv) Elevated alanine aminotransferase (v) Leukocytosis: white cell count >15,000/mm (vi) Normochromic, normocytic anaemia for age (vii) Sterile pyuria: >10 white blood cell/mm^3^

*If less than four of the principal features are present but two-dimensional echocardiography detects coronary artery abnormalities, patients are diagnosed with incomplete KD [2].

**Table 2:** Risk stratification based on degree of coronary artery involvement.

Risk stratification in Kawasaki disease [2, 27, 35, 44, 45]		
	Description	Recommended action
Level 1	No coronary artery changes detected at any stage	No intervention required beyond 8 weeks after illness onset

Level 2	Patient exhibits transient coronary dilation that regresses within 8 weeks	No intervention required beyond 8 weeks after illness onset

Level 3	Echocardiography locates an isolated small aneurysm in 1 coronary artery	(i) Long-term antiplatelet therapy with low-dose aspirin until regression (ii) Annual electrocardiogram (ECG) and echocardiograph (iii) Biennial stress test with myocardial perfusion imaging if child >10 years old (iv) Angiography if ischaemia is implicated

Level 4	Echocardiography locates at least one large coronary artery aneurysm	(i) Long-term antiplatelet therapy with low-dose aspirin (ii) Adjunctive warfarin or heparin therapy (iii) Biennial echocardiograph and ECG (iv) Annual stress test (v) Cardiac catheterisation with coronary angiography within 6–12 months and repeated if ischaemia is implicated

Level 5	Coronary artery involvement has progressed to coronary artery obstruction	(i) Long-term antiplatelet therapy with aspirin (ii) Adjunctive warfarin or heparin therapy (iii) Beta blockers to reduce myocardial oxygen consumption (iv) Biannual echocardiograph and ECG (v) Annual stress test
